# On Cholera, in Its Nature and Treatment

**Published:** 1854-07

**Authors:** Wm. Hanley

**Affiliations:** Naperville, Ill.


ART IV.—*On Cholera, its Nature and Treatment.* By Dr. WM. HANLEY, Naperville, Ill.

DEAR SIR,—I herewith lay before you some of my thoughts on the cause and cure of Asiatic Cholera. In the first place, I believe the sole cause to be a deficiency of saline in the blood; such deficiency being caused by excessive perspiration, together with increased temperature and moisture of the atmosphere. Soda being one of the chief constituents of bile, it is necessary that the blood should always be in a condition to supply the liver with a sufficiency, for none of a healthy character can be made without it. Liebig states that healthy blood within the body should contain one per cent. of saline. It is easy therefore to see that a deficiency of this property will produce disease. Water and alcoholic drinks are chiefly used in summer to quench thirst, neither of which contain saline enough to keep pace with the loss occasioned by diaphoresis and diuresis, in consequence of excessive quantities of such drinks. Every draught taken renders the blood less and less saline; hence deficiency ensues, and with it a non-formation of bile; non-formation of bile causes a non-neutralization of chyme, and a non-neutralization of chyme creates irritation of the lacteals, and such become relaxed, and excretients rather than absorbents. Not observing bile in the choleraic discharges, and an entire suppression of urine (generally), it is evident that some material is deficient in the blood, preventing both liver and kidneys from acting normally; and this material, in my opinion, is chloride of sodium.

I could lay before you a long paper on this subject, but my desire in this instance is to be brief. I will therefore state what method I have adopted for curing such cases of Asiatic cholera that have presented themselves to my notice, and I truly say with success in every instance of their use. I order the following at once:

| | | |
|---|---|---|
| ℞ | Hydra. sub. muriate, - | ℈j |
| | Chloride sodium, - | ℈j |
| | Musk, - - - | gr. ij. |
| | M. ft. xii. pulv. | |

Take one every 20 minutes *dry upon the tongue*, for the first hour; afterwards one every hour until a substance floats upon the surface of the discharges; then one every four hours until the whole are taken.

I give cold water for a drink, containing ʒj. chloride sodium to a quart. Should pain be felt in the epigastric region, I fold my handkerchief, wet a space with chloroform about the size of a two-shilling piece, and apply it to the surface of the body for four or five minutes, over where the pain is felt, and instant relief is consequent. When the discharges have ceased, I treat with light tonics, and order a nutritive diet

